# Herbal targeting of mitochondrial dynamic proteins in low-density lipoprotein driven atherosclerosis *in vitro*

**DOI:** 10.3389/fcvm.2026.1855659

**Published:** 2026-07-21

**Authors:** Zhanna Dzampaeva, Rodion Saveljev

**Affiliations:** Laboratory of System Ecological Analysis, North Ossetian State University, Vladikavkaz, Russia

**Keywords:** atherosclerosis, *Cynara cardunculus*, fission, fusion, HUVEC, inflammation, mitochondria, mitophagy

## Abstract

**Background:**

Mitochondrial dysfunction is a critical early driver of endothelial injury in atherogenesis. Natural plant-derived products targeting mitochondrial quality control represent a promising therapeutic approach, yet their mechanisms in oxidized LDL (ox-LDL)-induced endothelial damage remain underexplored.

**Methods:**

We evaluated ethanolic extracts from *Rosmarinus officinalis*, *Cynara cardunculus*, and *Myrtus communis* on ox-LDL-induced mitochondrial damage in human umbilical vein endothelial cells (HUVECs). Cell viability (MTT), proliferation (acridine orange staining), and mitochondrial membrane potential (Δψm; Rhodamine 6G) were assessed across therapeutic-equivalent concentrations (×0.1–×100). In a pathophysiologically relevant ox-LDL model (80 μg/mL, 48 h), RT-qPCR analyzed expression of genes regulating mitochondrial dynamics (DRP1, FIS1, MFF, MFN2, OPA1), mitophagy (PINK1, PARKIN, FUNDC1, OPTN, SQSTM1/p62), and antioxidant defense (CAT).

**Results:**

All three extracts preserved Δψm and mitochondrial network architecture in healthy HUVECs in a dose-dependent manner. In the ox-LDL model, extracts reversed the pro-fission transcriptional shift by downregulating DRP1, FIS1, and MFF while restoring MFN2 and OPA1 expression (*p* < 0.05–0.001). *C. cardunculus* robustly upregulated PINK1 and PARKIN, indicating enhanced ubiquitin-dependent mitophagy, whereas *R. officinalis* and *M. communis* preferentially increased FUNDC1 and OPTN, suggesting receptor-mediated clearance pathways. Extract treatment concurrently upregulated endogenous antioxidant enzymes (CAT), suppressed pro-inflammatory MAPK8 (JNK).

**Conclusions:**

Polyphenol-rich herbal extracts protect endothelial cells from ox-LDL-induced injury by rebalancing mitochondrial fission/fusion dynamics, activating complementary mitophagy pathways, and fortifying antioxidant defenses. These findings support the potential of standardized herbal formulations as targeted adjunctive strategies for early atherosclerosis prevention through mitochondrial quality control.

## 1 Introduction

Cardiovascular diseases (CVDs) remain the leading cause of global mortality worldwide. The number of deaths due to CVDs is expected to reach 23 million by 2030 (1). Atherosclerosis (AS) is the essential pathological basis underlying these conditions (2–4). Significant progress has been made over the last decade in revealing the molecular and cellular mechanisms of low-density lipoprotein (LDL) accumulation and pro-inflammatory activation of macrophages, endothelial cells, and vascular smooth muscle cells (VSMCs) (5, 6). Consequently, therapeutic approaches targeting key links in atherosclerosis pathogenesis—systemic inflammation, LDL, oxidative stress, and endothelial dysfunction—have been developed. However, researchers continue to investigate the early mechanisms initiating atherogenesis (7, 8). The first report linking mitochondria to atherosclerosis dates back to 1970 (8, 9). Mitochondrial dysfunction plays a key role in the pathogenesis of atherosclerosis, particularly in its early stages, as demonstrated by various *in vivo* and *in vitro* studies (4, 10, 11).

A healthy mitochondrial pool within the cell is maintained by a dynamic equilibrium between fission and fusion factors, modulated by specific proteins—dynamin-related protein 1 (DRP1), mitochondrial fission 1 protein (FIS1), mitofusin 1/2 (MFN1/2), and optic atrophy 1 (OPA1)—which sustain mitochondrial homeostasis. These proteins control the number of mitochondria in the cell and maintain a functional mtDNA pool; imbalance in these processes increases heteroplasmy of many known mtDNA mutations associated with atherosclerosis (8, 12–14). Mitophagy, in turn, modulates the response of macrophages, endothelial, and smooth muscle cells to various metabolic stressors and plays a fundamental role in their redox homeostasis and plasticity by degrading dysfunctional mitochondria (15, 16). The functional state of mitochondria in cells involved in atherogenesis, ensured by fission and fusion processes, plays a critical role in activating the systemic immune-inflammatory response, specifically in the differentiation of monocytes into type 1 macrophages (M1) within the subendothelial layer (8, 11, 17). Damaged and dysfunctional mitochondria are removed via mitophagy through pathways dependent on and independent of PTEN-induced putative kinase 1 (PINK1)/Parkin, mediated by proteins encoded by the PINK1, PARKIN, FUNDC1 genes (18, 19).

A critical and underexplored aspect is the potential molecular mechanisms of the effects of mitochondrial fission, fusion, and mitophagy proteins on systemic inflammation activation and atherogenesis (8, 20, 21). When mitochondrial fission excessively dominates over fusion (via Drp1 hyperactivation), the mitochondrial network becomes hyper fragmented. If this fragmented, damaged network cannot be cleared by efficient mitophagy, dysfunctional mitochondria accumulate within the endothelial cells. The oxidized LDL (oxLDL) accumulation in cells involved in atherogenesis, damages the mitochondria and directly causes a massive release of mitochondrial reactive oxygen species (mtROS) and oxidized mitochondrial DNA. Releasing mtROS that activate the NLR family pyrin domain containing 3 (NLRP3) inflammasome, thereby increasing levels of IL-1β and IL-18 (22, 23). This localized endothelial inflammation drives the expression of adhesion molecules, monocyte recruitment, and subendothelial lipid retention, ultimately initiating the atherosclerotic plaque.

The role of certain mitochondrial fission and fusion proteins and mitophagy in activating systemic inflammation and atherogenesis has been established (7, 24, 25). Drp1 is a protein involved in the fission of mitochondria and the endoplasmic reticulum. It is a key regulator of mitochondrial fission, necessary for maintaining the health and function of these organelles, and also regulates levels of mitochondrial reactive oxygen species (mtROS) and subsequent oxidative stress. In turn, ROS reduce the expression of OPA1, leading to remodeling of mitochondrial cristae and mitochondrial fragmentation (26, 27). Mfn 2 inhibits oxLDL-induced VSMC proliferation *in vivo* (28). Reduced levels of Mfn1, Mfn 2, and OPA1 lead to changes in mitochondrial membrane potential and damage. Mfn2 inhibits the PI3K/Akt pathway, activating mitochondrial apoptosis. Mfn2 deficiency leads to increased Ca^2^⁺ overload in cardiomyocytes (23).

Therefore, targeted therapy for mitochondrial dysfunction in atherosclerosis remains under investigation. Recently, global scientific interest in studying natural plant-derived products targeting mitochondrial function has increased, including the stimulation of biogenesis, regulation of mitochondrial fusion and fission, and mitophagy (29–31).

The selection of *Rosmarinus officinalis*, *Cynara cardunculus*, and *Myrtus communis* for this study was based on their rich profiles of bioactive polyphenols and diterpenes. *R. officinalis* is a valuable source of carnosic acid and rosmarinic acid. Carnosic acid is a lipophilic diterpene that has been shown to be a potent activator of the transcription factor Nrf2 (nuclear factor erythroid 2-related factor 2). Activation of Nrf2 triggers the expression of antioxidant enzymes (e.g., heme oxygenase-1) and, importantly for this study, promotes the degradation of damaged mitochondria (mitophagy) and the synthesis of new organelles (mitochondrial biogenesis) (32–34).

*Cynara cardunculus* is rich in unique caffeoylquinic acids and flavonoids, among which cynarin and luteolin deserve special attention. Cynarin is a key biomarker of artichoke, known for its ability to inhibit cholesterol biosynthesis and low-density lipoprotein (LDL) oxidation. The phenolic compounds of artichoke also modulate pro-inflammatory signaling pathways such as MAPK, which is critically important in the context of atherosclerotic inflammation (35).

*Myrtus communis* was chosen due to its high content of myricetin, a potent antioxidant flavonoid. In addition to its direct antioxidant effect, myrtle improves endothelial dysfunction and reduces oxidative stress in cardiac and aortic tissues under obese conditions, restoring the oxidant/antioxidant balance and enhancing the activity of endothelial nitric oxide synthase (eNOS) (36).

Based on this mechanistic cascade, we hypothesize a direct link wherein restoring mitochondrial quality control extinguishes the upstream triggers of endothelial inflammation and oxidative stress. Specifically, we postulate that these three herbal extracts protect HUVECs from ox-LDL-induced injury by normalizing mitochondrial dynamics (downregulating DRP1, FIS1, MFF and restoring MFN2, OPA1) and enhancing mitophagic clearance (via PINK1/PARKIN and receptor-mediated pathways). Concurrently, we hypothesize that the extracts reinforce cellular survival and redox defense by normalizing the pro-survival AKT pathway and upregulating key endogenous antioxidant enzymes, specifically superoxide dismutase (SOD) and catalase (CAT).

Thus, the primary objective of this study was to evaluate the effects of ethanolic extracts of *R. officinalis*, *C. cardunculus*, and *M. communis* on cell viability, proliferation, mitochondrial membrane potential, and the expression of genes regulating mitochondrial dynamics, mitophagy, inflammatory signaling, and antioxidant defense in an ox-LDL-induced *in vitro* atherosclerosis model.

## 2 Materials and methods

### 2.1 Cell culture and study design

Primary HUVEC cell line was obtained from N.N. Blokhin National Research Medical Center of Oncology of the Ministry of Health of the Russian Federation (Moscow). The HUVEC was maintained in Dulbecco's Modified Eagle's Medium (DMEM, PanEco, Russia) supplemented with 10% fetal bovine serum (FBS, PanEco, Russia), 1% penicillin/streptomycin (PanEco, Russia). The HUVEC cells used in the present study were from the early passages (passages 2–4) (37). Cells were incubated at 37 °C in a humidified atmosphere containing 5% carbon dioxide (CO_2_). The medium was changed every 2 days to maintain optimal cell growth. When the cells reached 70%–80% confluence in the culture flask, they were digested with 0.25% trypsin-EDTA solution (PanEco, Russia), collected, and centrifuged. HUVECs (1.5 × 10^4^ cells/well) were plated onto 96-well cell culture plates for future experiments: cell viability, proliferation, and mitochondrial activity assays. After 24, 48, and 72 h of incubation, images were captured using an EVOS M 7000 microscope and its software (Thermo Fisher Scientific, USA).

Experimental design:

1. Functional assays of extracts on healthy HUVECs (сell Viability Assay (MTT), proliferation, Mitochondrial Activity Assay [Rhodamine 6G staining, mitochondrial fragmentation index (MFI)];
2. Molecular assays [real time—quantitative real time-PCR (RT-qPCR)]—ox-LDL model of atherosclerosis treated with extract (5 groups)—herbal extracts effects on the expression of mitochondrial dynamics proteins:
  1. control (HUVEC)—intact cells;
  2. HUVEC + ox-LDL (80 μg/mL) for 48 h;
  3. HUVEC + ox-LDL (80 μg/mL) + *Rosmarinus officinalis* (×1) for 48 h;
  4. HUVEC + ox-LDL (80 μg/mL) + *Myrtus communis* (×1) for 48 h;
  5. HUVEC + ox-LDL (80 μg/mL) + *Cynara cardunculus* (×1) for 48 h.

To ensure assay validity and prevent artifacts, all fluorometric assays (Rhodamine 6G, Acridine Orange) were validated for phototoxicity by confirming that laser exposure times (<200 ms) did not alter baseline Δψm. All functional and molecular assays were performed with a minimum of *n* = 3 independent biological replicates (distinct cell passages) and *n* = 3 technical replicates for molecular assays per experiment.

### 2.2 Preparation and characterization of herbal extracts

A conventional Soxhlet extraction apparatus, consisting of a condenser, a Soxhlet chamber, and an extraction flask, was used. Dried and ground *Cynara cardunculus*, *Rosmarinus officinalis*, *Myrtus communis* were placed in the Soxhlet apparatus and extracted with ethanol for 7 h (38). The ratio of herbal material to solvent is presented in Table 1.

**Table 1:** Quantitative ratio of dried herbal material (mg) to 1 mL 70% ethanol.

Herbal materials	Concentration (mg/mL)
*Rosmarínus officinalis*	34.8
*Cynara cardunculus*	35.3
*Myrtus communis*	10.5

The solvent was then completely removed under reduced pressure at 50 °C to obtain dry residues. These dry extracts were reconstituted directly in cell culture medium (DMEM supplemented with 10% FBS and 1% penicillin/streptomycin) to prepare stock solutions.

For all extracts, a single therapeutic dose of 2 g per 70 kg of human body weight (28.60 μg/mL of medium) was adopted, which corresponds to the average therapeutic dose in clinical phytotherapy. For *in vitro* experiments, the stock solution (2,860 μg/mL, denoted as ×100) was serially diluted with culture medium to obtain a series of working concentrations: ×50, ×25, ×10, ×2.5, ×1, and ×0.1. This dosing strategy allows for the evaluation of the extracts' effects across a wide dynamic range—from subtherapeutic (×0.1) to supratherapeutic (×100) levels—enabling the identification of dose-dependent relationships and the determination of optimal therapeutic windows for each herbal preparation.

Phytochemical profiling of *Cynara cardunculus*, *Rosmarinus officinalis*, *Myrtus communis* was performed using the Ultimate 3000 liquid chromatography system, equipped with a degasser, a three-chamber pump, a column thermostat, a thermostatic autosampler, a diode-matrix spectrophotometric detector (DMD) and a triple quadrupole mass spectrometric detector (MS) (HPLC-DAD-MS) TSQ Endura (Thermo Scientific, USA). HPLC conditions: stationary phase—column Waters NovaPak® C18 ID 4 µm 4.6 mm × 150 mm; mobile phase A—0.1% aqueous solution of formic acid, B—acetonitrile; flow rate of mobile phase 0.5 mL/min; gradient elution 0–30 min 10%–25% V, 30–40 min 25%–50% V, column regeneration 40–45 min 50%–10% V, 45–50 min 10% V; column temperature 25 °C; volume of the injected sample 10 µL; autosampler temperature 20 °C; detection was performed at four values of analytical wavelengths—370, 350, 338 and 290 nm.

### 2.3 Cell viability assay (MTT)

To evaluate the effect of herbal extracts on the viability of HUVECs, an MTT assay was performed. HUVECs were plated into 96-well plates at a density of 1.5 × 10^4^ cells/well and incubated in 100 μL (DMEM, PanEco, Russia) supplemented with 10% fetal bovine serum (FBS, PanEco, Russia), 1% penicillin/streptomycin (PanEco, Russia). Cells were incubated at 37 °C in a humidified atmosphere containing 5% carbon dioxide (CO_2_) to 50%–60% confluence with serial dilutions of herbal extracts 24 h. After 50 μL of nitrotetrazolium blue chloride solution (Sigma-Aldrich) (5 mg/mL) were added to each well and incubated for 4 h at 37 °C in the dark. The MTT formazan crystals were dissolved in 200 μL of DMSO added to each well, and the plates were shaken for 10 min. The optical density was measured at 580 nm. Cell viability was expressed as a percentage and calculated by comparing the absorbance of treated cells vs. untreated control cells (37).

### 2.4 Cell proliferation assay

HUVEC cells (1.5 × 10^4^ cells/well) were plated into 96—well culture plates. Cells were incubated at 37 °C in a humidified atmosphere containing 5% carbon dioxide (CO_2_) for 24 h. Cells were cultured in appropriate media, and after reaching 50% confluency, the test herbal extracts were added at the required concentrations [sub-therapeutic (×0.1), therapeutic (×1) to supra-therapeutic (×2.5) levels] of herbal extracts. After 24 and 48 h of incubation, cells were stained with acridine orange. Sixty images per well were captured using an EVOS M7000 microscope (Thermo Fisher Scientific, USA). Cell proliferation data were analyzed using EVOS analysis software (Thermo Fisher Scientific, USA) (37).

### 2.5 Mitochondrial activity assay

Mitochondrial membrane potential (Δψm) is a major component of the proton motive force (mitochondrial electrochemical transmembrane potential), which plays a key role in mitochondrial bioenergetics, metabolic, and signaling functions (39). HUVEC cells (1.5 × 10^4^ cells/well) were plated into 96-well culture plates and incubated at 37 °C in a humidified atmosphere containing 5% CO_2_ for 24 h. After reaching 40% confluency, the test herbal extracts were added at the required concentrations. After 24 and 48 h of incubation, cells were stained with Rhodamine 6G (20 nM). Sixty images per well were captured using an EVOS M7000 microscope (Thermo Fisher Scientific, USA), data were analyzed using EVOS analysis software (Thermo Fisher Scientific, USA).

Additionally, the mitochondrial fragmentation index (MFI) was calculated by counting non-contiguous mitochondrial particles and dividing by the number of pixels comprising the mitochondrial network. To ensure objective quantification, images were converted to 8-bit grayscale. Background subtraction (rolling ball radius = 50 pixels) and global thresholding (Otsu method) were applied uniformly across all groups. Discrete mitochondrial particles were defined as objects between 0.5 and 10 μm^2^; objects smaller than 0.5 μm^2^ (background noise) or larger than 10 μm^2^ (intact networks) were excluded from the count. MFI = (number of discrete mitochondrial segments/total mitochondrial mass in black pixels) × 1,000. A higher MFI corresponds to a more fragmented network (40). Mitochondrial activity assay data were analyzed using EVOS software (Thermo Fisher Scientific, USA).

### 2.6 *In vitro* model of atherosclerosis

LDL were collected from blood samples of healthy donors following informed consent acquisition. Blood samples were collected without anticoagulants and centrifuged at 1,500 × g for 10 min. The resulting supernatant (serum) was pooled into a 50 mL tube. Subsequently, 5 mL of serum were added to 45 mL of a sterile precipitating solution containing sodium citrate (0.064 mmol/L) and heparin (50 IU), with the pH adjusted to 5.1 using a 5M HCl solution. The mixture was incubated for 10 min at 20 °C. LDL was then precipitated by centrifugation at 1,000 × g for 10 min at 20 °C, ensuring that no heating of the mixture occurred. The isolated LDL were subsequently oxidized by incubation with 100 µM H_2_O_2_ for 24 h at 37 °C. The degree of oxidation was confirmed by measuring thiobarbituric acid reactive substances (TBARS). This procedure yielded the requisite quantity of ox-LDL for subsequent investigation (25).

Based on the effects on cell viability, migration, proliferation, and mitochondrial activity, the most effective herbal extracts were selected for future experiments on an *in vitro* model of atherosclerosis.

HUVECs were stimulated by oxidized low-density lipoproteins by 80 μg/mL concentration for 48 h to induce an *in vitro* atherosclerosis model (41). After that the ×1 concentrations of *Cynara cardunculus*, *Rosmarinus officinalis*, *Myrtus communis* dry extract were added to medium and incubated for 48 h.

Lipid accumulation in HUVECs was detected using Oil-red O staining. Briefly, cell culture medium was carefully removed and HUVECs were fixed with 10% formaldehyde for 30 min. Subsequently, HUVECs were stained with Oil-red O staining for 10 min. In addition, cell nuclei were stained with 5 μg/μL Hoechst 33342 for 10 min. Finally, the oil-red-positive cells were observed and images were captured at 10 randomly selected fields under a microscope and analyzed by EVOS software (Thermo Fisher Scientific, USA).

### 2.7 Quantitative real time-PCR

Total RNA from HUVECs was extracted using ExtractRNA reagent following the manufacturer's instructions (Evrogen, Moscow, Russia). RNA purity was assessed using a NanoPhotometer NP 80 (IMPLEN, Germany). The RNA purity was based on the A260/A280 ratio, with values between 1.7 and 2.0 indicating high-quality RNA. Real-time quantitative reverse transcription polymerase chain reaction (RT-qPCR) was conducted using 5X qPCRmix-HS SYBR containing HS Taq DNA polymerase, SYBR Green I dye, a mixture of deoxynucleoside triphosphates, Mg^2^⁺, and a reaction buffer (Evrogen, Moscow, Russia) on a QuantStudio 5 Dx real-time PCR system (Thermo Fisher Scientific, Waltham, MA, USA). To ensure high specificity and avoid genomic DNA amplification, all primers were designed by The National Center for Biotechnology Information (https://www.ncbi.nlm.nih.gov/), IDT Technologies (https://eu.idtdna.com/pages/products/qpcr-and-pcr/gene-expression/primetime-primer-only-assays) and synthesized by Evrogen (Moscow, Russia) to span exon-exon junctions and were pre-validated for amplification efficiency (90%–110%) and specificity. Crucially, following each RT-qPCR run, a melting curve analysis was systematically performed to confirm the amplification of a single, specific PCR product and to rule out the presence of primer-dimers or non-specific amplification artifacts. The quantitative real-time PCR was performed according to standard protocols. The average cycle thresholds (Ct) were calculated using the 2^⁻ΔΔCt^ method to determine relative gene expression levels (40, 42), normalized to GAPDH, and analyzed using Thermo Fisher Connect Software (Thermo Fisher Scientific, Waltham, MA, USA). Primer sequences are provided in Table 2.

**Table 2:** Primer sequences for real-time quantitative PCR.

Gene	Forward primers (5′-3′)	Reverse primers (5′-3′)
MFN2	CCTTCCTTGAAGACACGTACAG	GATGCCTCTCACTTTGGATAGG
OPA1	GAGATCAGAGTGCTGGAAAGAC	CAGAGTCACCTTAACTGGAGAAC
DRP1	AATGACCAAGGTGCCTGTAG	GCAGTGACAGCGAGGATAAT
FIS1	CAGCGGGATTACGTCTTCTAC	TTCCTTGGCCTGGTTGTT
MFF	ACAACTTCTGGAAGAGGAGAAC	CGAAACCAGAGCCAGCTATTA
PINK1	GTCAGGAGATCCAGGCAATTT	CTTACCAATGGACTGCCCTATC
PARKIN	AACCGGATGAGTGGTGAATG	AGCTACTGGTGTTTCCTTGTC
FUNDC1	AGTTCGGGACCTATGGTAGAA	AAGAAGAAAGCCACCACCTAC
OPTN	CAAGCCATGAAAGGGAGATTTG	AAGGCCATTAGACGCTCTTT
SQSTM1/p62	GGAACAGATGGAGTCGGATAAC	ATCTGTAGGGACTGGAGTTCA
CAT	CTCCACTGTTGCTGGAGAAT	CGAGATCCCAGTTACCATCTTC
SOD	GTGTGGCCGATGTGTCTATT	CACCTTTGCCCAAGTCATCT
AKT	GCACCAGAGGTGTTAGAAGATAA	TCATGGTCCTGGTTGTAGAAAG
MAPK8	CCACCAAAGATCCCTGACAA	GTTCTCTCCTCCAAGTCCATAAC
GAPDH	GTCAACGGATTTGGTCGTATTG	TGTAGTTGAGGTCAATGAAGGG

MFN1/2, mitofusin 1/2; OPA1, optic atrophy 1; DRP1, dynamin-related protein 1; FIS1, mitochondrial fission protein 1; MFF, mitochondrial fission factor; PINK1, PTEN-induced putative kinase 1; PARKIN, Parkin RBR E3 ubiquitin protein ligase; FUNDC1, FUN14 domain containing 1; OPTN, optineurin; SQSTM1/p62, sequestosome 1; MAPK8, JNK—mitogen-activated protein kinase 8; CAT, catalase; SOD, superoxide dismutase; AKT, protein kinase B; GAPDH, glyceraldehyde-3-phosphate dehydrogenase.

### 2.8 Statistical analysis

GraphPad Prism 7 software was used to show all data reports (GraphPad, Inc., La Jolla, CA, USA). All data were assessed for normality of distribution using the Shapiro–Wilk test. All values are expressed as mean ± standard deviation (SD). A Kruskal–Wallis one-way analysis of variance (non-parametric ANOVA) on ranks with a *post hoc* Dunn's test was performed to evaluate the differences between experimental groups. *P* < 0.05 was considered statistically significant.

## 3 Results

### 3.1 Phytochemical profiling of herbal extracts

HPLC-DAD-MS analysis confirmed the presence of the targeted marker compounds. *R. officinalis* extract 20.7 mg/dm^3^ of rosmarinic acid. *C. cardunculus* was standardized to 34.48 mg/dm^3^ of cynarin, *M. communis* contained 2,963 mg/dm^3^ of myricetin. Batch-to-batch reproducibility analysis across three independent extractions demonstrated high consistency for all marker compounds. In Table 3 are presented results of HPLC-DAD-MS for the extracts, which were used in this experiment with HUVEC.

**Table 3:** HPLC-DAD-MS results for *Cynara cardunculus*, *Rosmarinus officinalis*, *Myrtus communis*.

Extract	Major marker compounds	*R_t_*, min	Concentration, mg/dm^3^	Primary biological activity in literature
*Rosmarinus officinalis*	Rosmarinic acid	20.7	535	Nrf2 activation, antioxidant, mitophagy induction
	Rutin	17.4	255	ROS scavenging, vascular protection
	Ferulic acid	17.8	120	Anti-inflammatory, antioxidant
	Caffeic acid	13.9	2	Antioxidant
*Cynara cardunculus*	Chlorogenic acid	13.7	57.14	LDL oxidation inhibition
	Cynarin (1,5-di-O-caffeoylquinic acid)	29.6	34.48	Cholesterol biosynthesis inhibition
*Myrtus communis*	Myricetin-3-O-rhamnoside (myricitrin)	17.1	2,963.0	Endothelial protection, eNOS activation
	Myricetin-3-O-galactoside	15.8	704.7	Antioxidant, redox balance

Nrf2, nuclear factor erythroid 2-related factor 2; ROS, reactive oxygen species; LDL, low-density lipoprotein; eNOS, endothelial nitric oxide synthase.

### 3.2 Effect of herbal extracts on HUVEC viability

To determine the safe working concentrations of the herbal extracts, cell viability was assessed using the MTT assay. HUVECs were treated with varying concentrations of *Rosmarinus officinalis*, *Cynara cardunculus*, *Myrtus communis* extracts. The half-maximal inhibitory concentration (IC 50) was calculated for each extract to establish the therapeutic window. As presented in Table 4, *M. communis* exhibited the lowest IC 50 value (328.60 μg/mL), indicating higher potency compared to *Rosmarinus officinalis L.* (454.09 μg/mL) and *Cynara cardunculus* (540.79 μg/mL). Based on these results, concentrations below the IC 50 values were selected for subsequent functional assays to ensure that observed effects were not due to cytotoxicity.

**Table 4:** Cell viability assay results (IC 50 values).

Herbal extract	IC 50 (µg/mL)
*Rosmarínus officinalis*	454.09
*Cynara cardunculus*	540.79
*Myrtus communis*	328.60

### 3.3 Dose-dependent modulation of HUVEC proliferation

**Figure 1:**
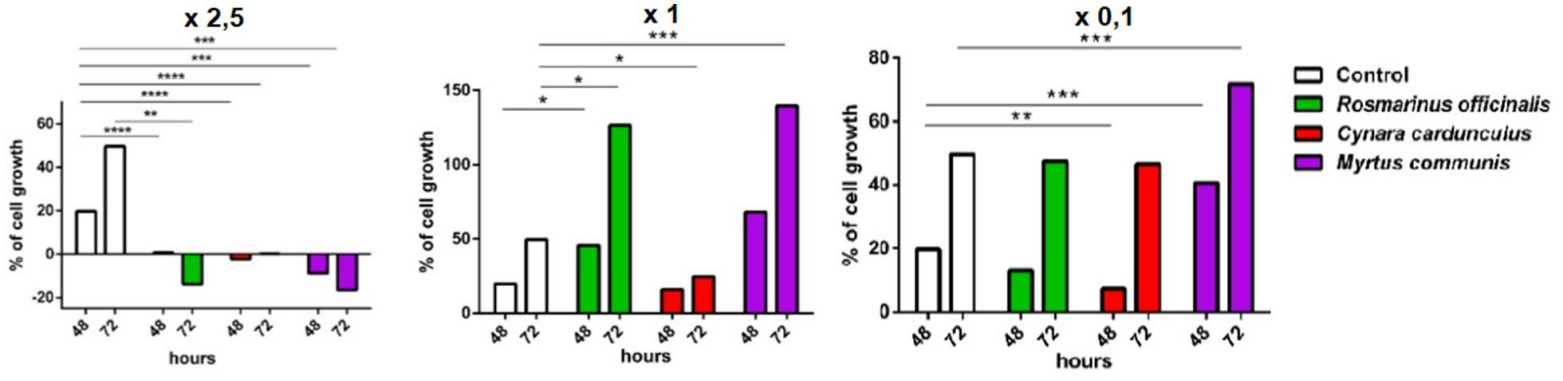
Quantitative analysis of cell proliferation (% of untreated control) at 48 and 72 h after treatment with ×2.5, ×1, ×0.1 equivalent concentrations of *R. officinalis* and *M. communis*, *C. cardunculus* extracts. Data are presented as mean ± standard deviation (SD). *p < 0.05, **p < 0.01, ***p < 0.001, ****p < 0.0001 vs. control (Kruskal–Wallis test).

Proliferation was evaluated by acridine orange staining after 48 and 72 h of treatment with extracts at ×0.1, ×1, and ×2.5 equivalents (2.86, 28.6, and 71.5 µg/mL, respectively). As shown in Figure 1, at the highest tested concentration (×2.5), all three extracts caused a reduction in HUVEC proliferation compared to the untreated control after 48 h (*p* < 0.05). At therapeutic (×1) and sub therapeutic (× 0.1) concentrations, significantly increasing effect on cell growth was observed relative to control (*p* > 0.05), especially, *M. communis.* These findings confirm that therapeutic and sub-therapeutic doses preserve endothelial replicative capacity, whereas supra-therapeutic concentrations exert mild growth-restrictive effects.

### 3.4 Restoration of mitochondrial membrane potential

**Figure 2:**
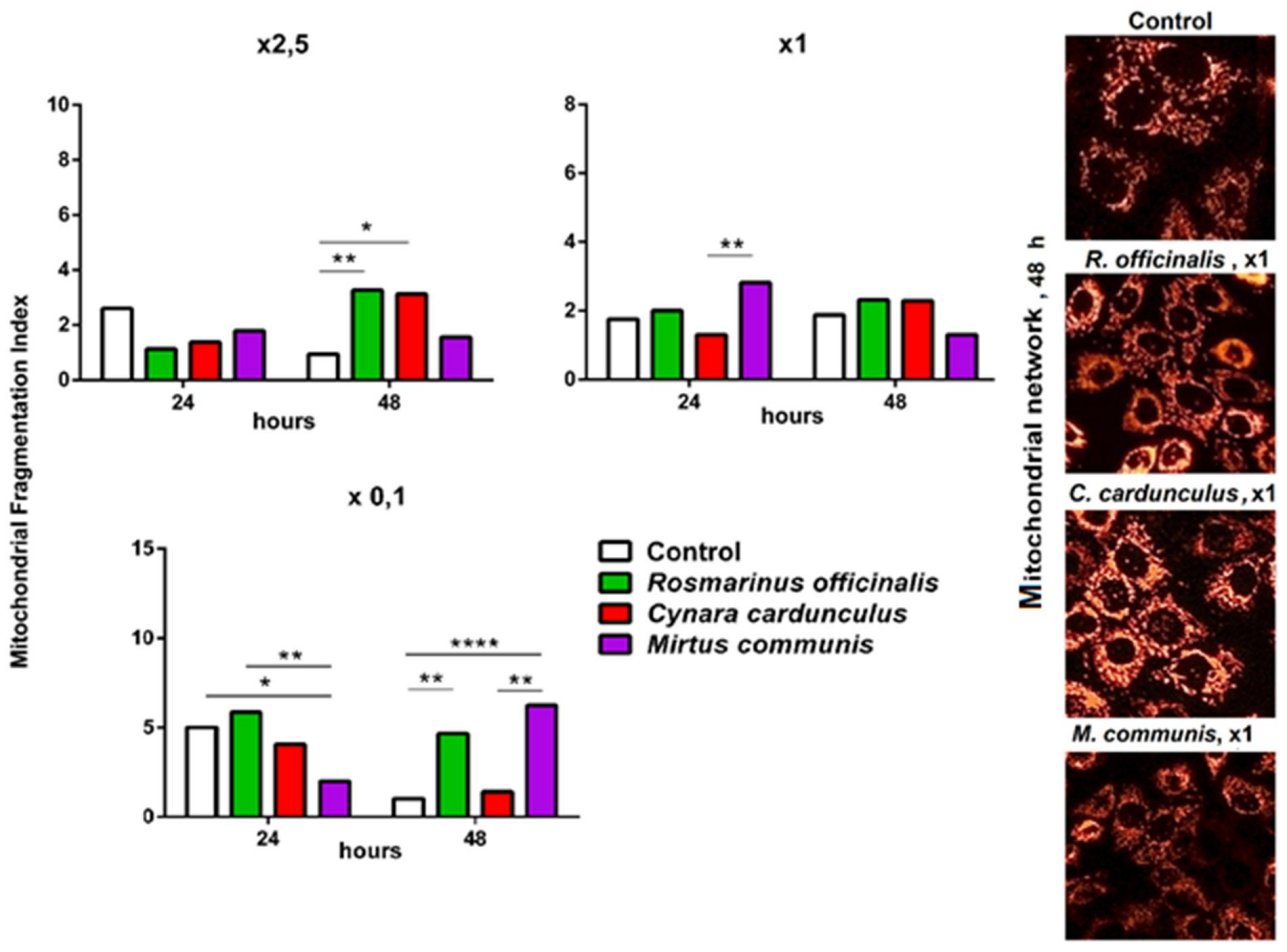
Mitochondrial fragmentation index (MFI) at 24 and 48 h after treatment with ×2.5, ×1, and ×0.1 equivalent concentrations of herbal extracts. **p < 0.01, ***p < 0.001, *p* < 0.0001 vs. control (Kruskal–Wallis test).

Mitochondrial membrane potential (Δψm) was evaluated using Rhodamine 6G staining to assess mitochondrial health. Treatment with the herbal extracts preserved Δψm in a dose-dependent manner. Treatment with all three extracts at ×2.5 concentration had a tendency (*p* ≥ 0.05) to reduced mitochondrial fragmentation index (MFI) compared to control after 24 h of incubation, then after 48 h of treatment *R. officinalis* (*p* < 0.001) and *M. communis* (*p* < 0.01) increased MFI compared to control (Figure 2). No effect on MFI compared to control was seen at × 1 (Figure 2).

At × 0.1 concentration, *M. communis* showed a significant reduction of MFI (*p* < 0.005) after 24 h. After 48 h of treatment *R. officinalis* (*p* < 0.01) and *M. communis* (*p* < 0.001) increased MFI compared to control (Figure 2).

**Figure 3:**
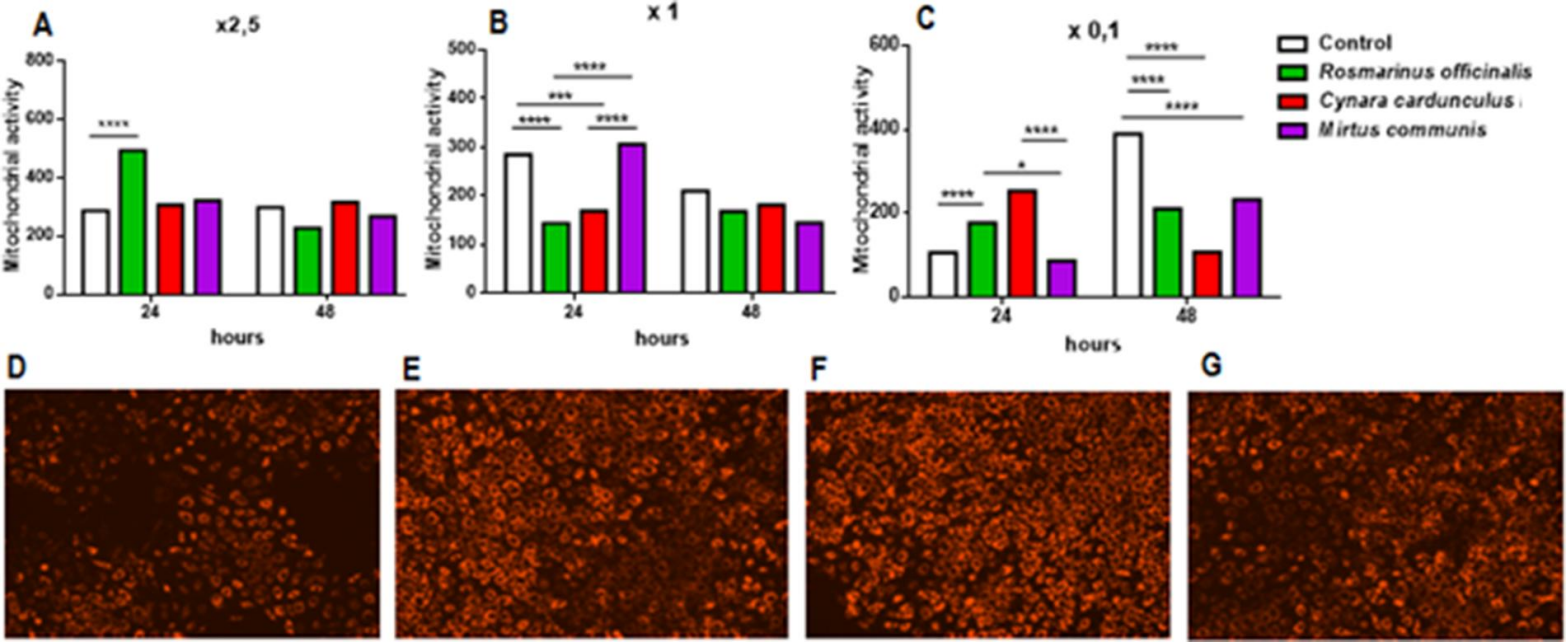
**(A–C)** Mitochondrial activity in HUVECs treated with herbal extracts. Data are mean ± SD. *p < 0.05, **p < 0.01, ***p < 0.001, *p* < 0.0001 vs. control. **(D–G)** Representative images of mitochondrial staining (Rhodamine 6G): **(D)** control, **(E)** *Rosmarinus officinalis*, ×1, **(F)** *Myrtus communis*, ×1, **(G)** *Cynara cardunculus*, ×1. Mitochondrial health was assessed on healthy HUVECs exposed to herbal extracts alone, using Rhodamine 6G staining to evaluate Δψm. Data were analyzed using EVOS analysis software (Thermo Fisher Scientific, USA) (×20).

All three extracts preserved Δψm in a dose-dependent manner. At ×1 and ×0.1 concentration, Δψm remained comparable to control. At ×2.5, a slight but non-significant decrease in fluorescence intensity was observed for *R. officinalis* and *C. cardunculus* (*p* > 0.05), while *M. communis* maintained Δψm at control levels, suggesting superior mitochondrial stabilization at higher doses (Figures 3A–C).

Representative fluorescence images (Figures 3D–G) corroborated quantitative data, showing preserved reticular mitochondrial architecture in extract-treated groups [*Rosmarinus officinalis*, *×*1 (E), *Myrtus communis*, ×1 (F), *Cynara cardunculus*, ×1 (G)] compared to control to the more fragmented pattern occasionally observed at higher doses. These results demonstrate that the extracts exert direct, dose-dependent modulatory effects on mitochondrial dynamics in healthy endothelial cells, independent of oxidative stress (Figures 3D–G).

### 3.5 Herbal extracts reverse ox-LDL-induced dysregulation of mitochondrial dynamics and quality control genes

**Figure 4:**
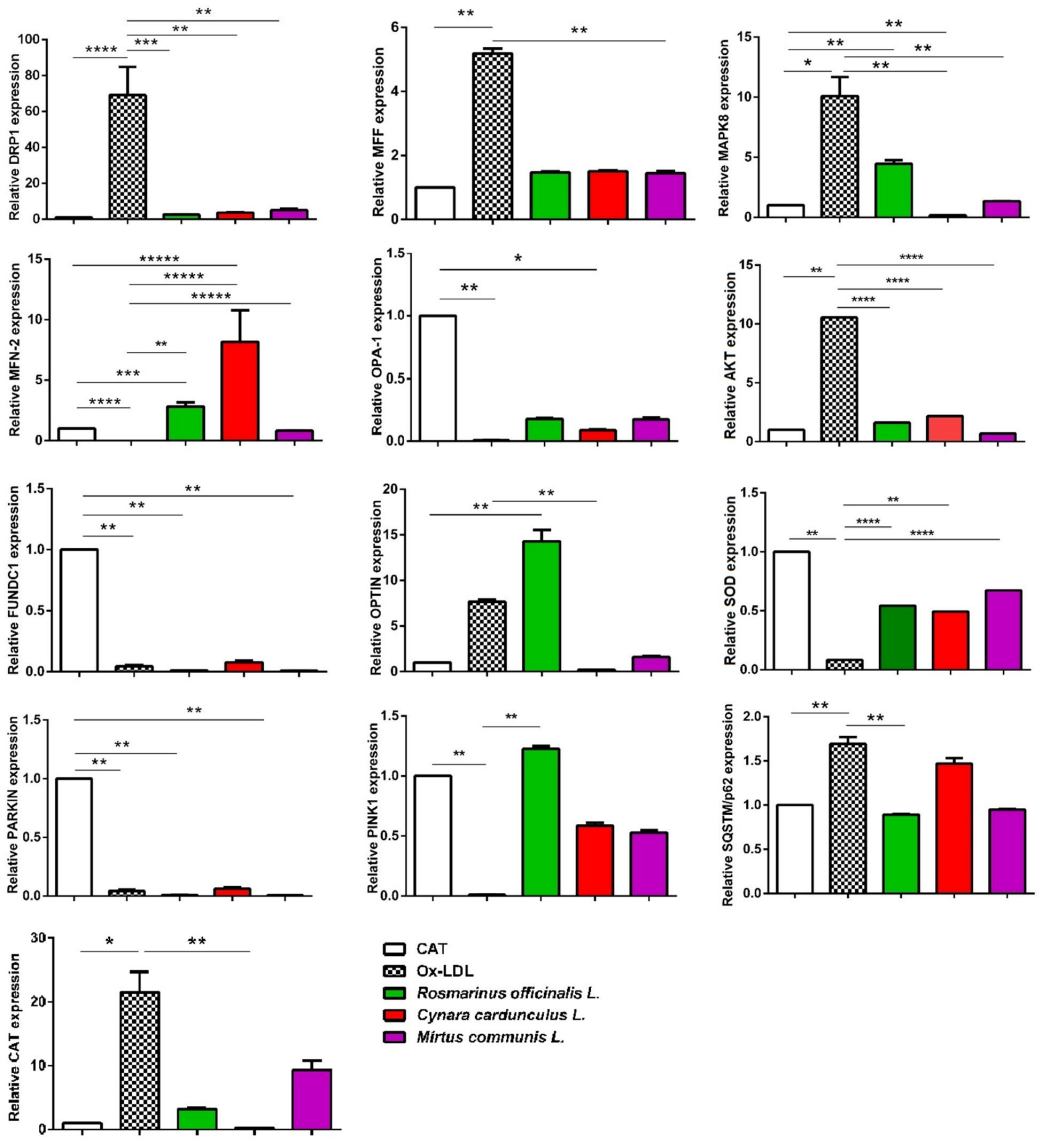
Mitochondrial fission, fusion, and mitophagy proteins genes expression results (RT-qPCR) across five experimental groups: (1) control; (2) ox-LDL (80 μg/mL for 48 h); (3) *Rosmarinus officinalis;* (4) *Myrtus communis;* (5) *Cynara cardunculus* MFN2 (mitofusin 2); OPA1 (optic atrophy 1); DRP1 (dynamin-related protein 1); FIS1 (mitochondrial fission protein 1); MFF (mitochondrial fission factor); PINK1 (PTEN-induced putative kinase 1); PARKIN (parkin RBR E3 ubiquitin protein ligase); FUNDC1 (FUN14 domain containing 1); OPTN (optineurin); SQSTM1/p62 (sequestosome 1); MAPK8 (JNK)—mitogen-activated protein kinase 8; SOD (superoxide dismutase); AKT (protein kinase B); CAT (catalase); GAPDH (glyceraldehyde-3-phosphate dehydrogenase).

To evaluate protective mechanisms in a pathophysiologically relevant context, HUVECs were co-incubated with extracts at ×1 equivalent (28.6 μg/mL) and ox-LDL (80 μg/mL) for 48 h. To elucidate the molecular mechanisms underlying the protective effects, we analyzed the expression of genes involved in mitochondrial fission, fusion, and mitophagy using RT-qPCR across five experimental groups: (1) control; (2) ox-LDL; (3) *Rosmarinus officinalis;* (4) *Myrtus communis;* (5) *Cynara cardunculus*.

ox-LDL exposure significantly upregulated mitochondrial fission (DRP1, FIS1, MFF; *p* < 0.01 vs. control) and downregulated fusion proteins (MFN2, OPA1; *p* < 0.05). All three extracts reversed this dysregulation (Figure 4).

*R. officinalis* most potently suppressed DRP1 expression (2.3-fold reduction vs. ox-LDL, *p* < 0.001) and restored MFN2 to near-baseline levels (1.9-fold upregulation, *p* < 0.01). *M. communis* demonstrated balanced modulation, concurrently downregulating DRP1 and FIS1 while enhancing OPA1 expression. *C. cardunculus* showed moderate but consistent restoration of fusion markers without significantly altering fission gene transcription (Figure 4).

ox-LDL impaired expression of mitophagy genes (PINK1, PARKIN, FUNDC1, OPTN, SQSTM1/p62), suggesting flux blockade. Herbal treatment differentially restored these pathways. C. cardunculus uniquely and robustly upregulated PINK1 (3.1-fold) and PARKIN (2.7-fold) vs. ox-LDL (*p* < 0.01), indicating enhanced PINK1/Parkin-dependent mitophagic flux. *R. officinalis* and *M. communis* moderately increased FUNDC1 and OPTN expression, pointing to complementary, receptor-mediated mitophagy activation (Figure 4).

Consistent with improved mitochondrial function, extract treatment upregulated endogenous antioxidant enzymes: *M. communis* significantly increased CAT expression (*p* < 0.05).

ox-LDL exposure (80 μg/mL, 48 h) caused a dramatic suppression of SOD expression, reducing (*p* < 0.0001 vs. control). Treatment with all three extracts significantly restored SOD expression, with *M. communis* showing the strongest recovery (*p* < 0.01 vs. ox-LDL).

Conversely, ox-LDL induced an upregulation of AKT expression, reaching nearly 10-fold higher levels than the control (*p* < 0.0001). This aberrant overexpression of AKT, a key pro-survival kinase, often reflects cellular stress and compensatory signaling. All three extracts effectively normalized AKT expression, reducing it to approximately 2–3 fold over control levels (*p* < 0.001 vs. ox-LDL), suggesting a restoration of balanced survival signaling.

**Figure 5:**
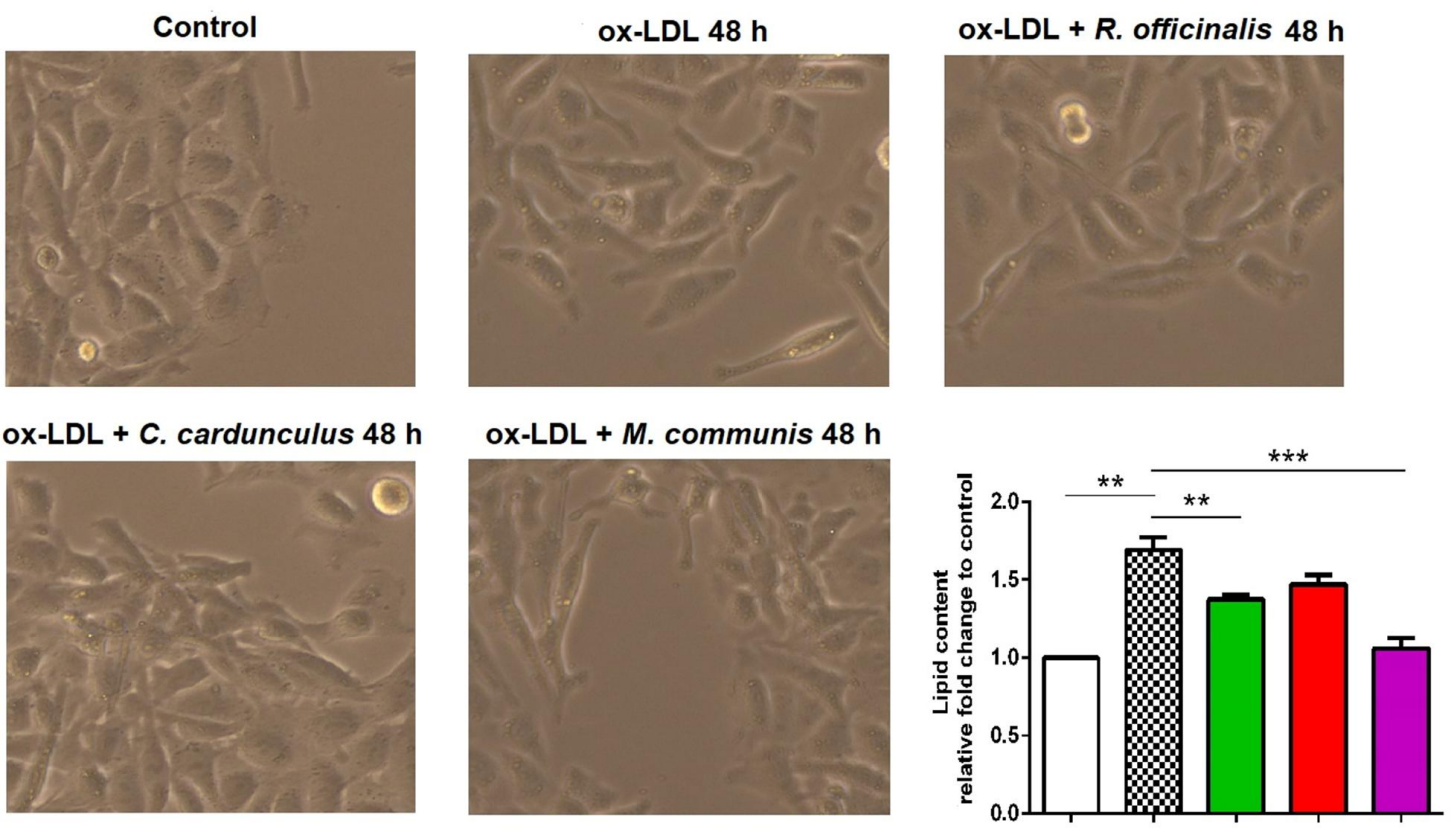
Results of herbal treatment of *in vitro* model of atherosclerosis in experimental groups: **(A)** control (HUVEC); **(B)** HUVEC + ox-LDL (80 μg/mL for 48 h; **(C)** HUVEC + ox-LDL (80 μg/mL) + *Rosmarinus officinalis* (×1) for 48 h; **(D)** HUVEC + ox-LDL (80 μg/mL) + *Myrtus communis* (×1) for 48 h; **(E)** HUVEC + ox-LDL (80 μg/mL) + *Cynara cardunculus* (×1) for 48 h. Cells morphology was analyzed using EVOS analysis software (Thermo Fisher Scientific, USA) (×20).

Furthermore, pro-inflammatory MAPK8 (JNK) was significantly downregulated (*p* < 0.05), suggesting reduced oxidative stress signaling (Figure 4). The morphology HUVEC of all experimental groups is presented in Figure 5(A-E).

## 4 Discussion

The accumulation of oxidized low-density lipoprotein (ox-LDL) in endothelial cells is a critical initiating event in atherogenesis (43–45). In *in vitro* model (80 μg/mL, 48 h), ox-LDL induced a profound transcriptional dysregulation of mitochondrial dynamics. Specifically, we observed a >20-fold upregulation of DRP1 (dynamin-related protein 1) and significant increases in FIS1 and MFF (*p* < 0.01). DRP1 is the master effector of mitochondrial fission; upon activation, it translocates to the outer mitochondrial membrane, where FIS1 and MFF serve as recruitment factors. Physiologically, controlled fission is essential for isolating damaged mitochondrial segments. However, the excessive DRP1 activity observed here leads to hyper-fragmentation of the mitochondrial network, a hallmark of bioenergetic failure and a precursor to mtROS overproduction (26, 27).

Concurrently, ox-LDL suppressed the expression of fusion genes—MFN2 (mitofusin 2) and OPA1 (optic atrophy 1). MFN2 mediates outer membrane fusion, while OPA1 regulates inner membrane fusion and cristae architecture. Reduced MFN2 not only impairs mitochondrial network connectivity but also disrupts calcium homeostasis and sensitizes cells to apoptosis via the PI3K/Akt pathway (23, 28, 46). The resulting imbalance—fission dominating over fusion—creates a dysfunctional mitochondrial pool that fails to meet cellular energy demands and serves as a source of damaging mtROS and oxidized mtDNA.

**Figure 6:**
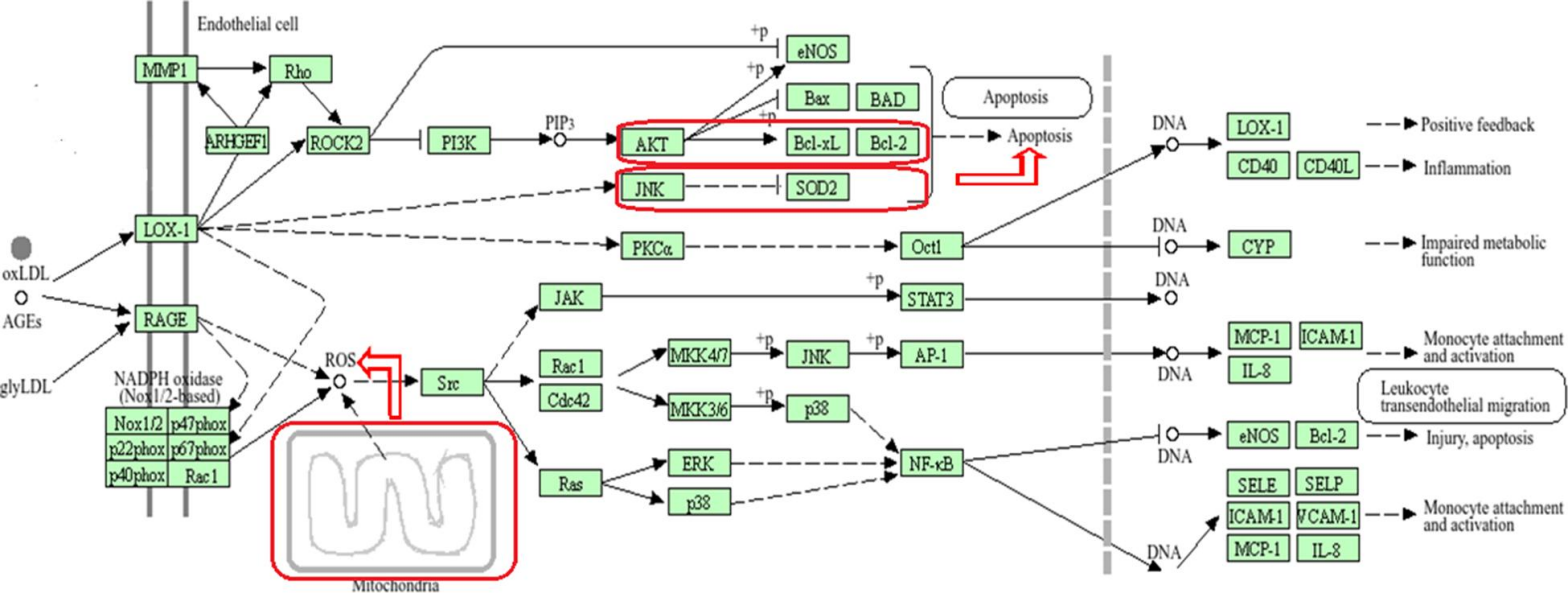
Schematic representation of the proposed molecular mechanisms underlying the protective effects of *Rosmarinus officinalis*, *Cynara cardunculus*, and *Myrtus communis* ethanolic extracts against ox-LDL-induced endothelial dysfunction. The diagram illustrates the sequential pathogenic cascade initiated by ox-LDL accumulation, including mitochondrial fission/fusion imbalance, impaired mitophagy, mtROS overproduction, and subsequent NF-*κ*B-driven inflammation. The central panel depicts the multi-target intervention of the studied extracts: (1) normalization of mitochondrial dynamics; (2) reinforcement of endogenous antioxidant defenses (SOD, CAT); (3) modulation of survival and inflammatory signaling (AKT normalization, MAPK8/JNK suppression). Solid arrows indicate experimentally validated transcriptional changes observed in this study; dashed arrows represent hypothesized downstream effects requiring further investigation. KEGG PATHWAY Database was used in the creation of this figure (https://www.kegg.jp/kegg/pathway.html).

The integrated molecular mechanisms underlying these protective effects, linking mitochondrial quality control to the attenuation of endothelial inflammation, are summarized in the schematic diagram (Figure 6).

All three herbal extracts reversed the ox-LDL-induced fission/fusion imbalance, but through distinct molecular mechanisms. *Rosmarinus officinalis* most potently suppressed DRP1 expression (2.3-fold reduction vs. ox-LDL, *p* < 0.001) and restored MFN2 levels (1.9-fold upregulation). Carnosic acid, a major diterpene in *R. officinalis*, is a known activator of Nrf2 (nuclear factor erythroid 2-related factor 2). Nrf2 activation can transcriptionally repress DRP1 and promote MFN2 expression, potentially via antioxidant response elements (AREs) or through indirect stabilization of OPA1-dependent cristae integrity (32–34).

*Myrtus communis* demonstrated balanced modulation: it concurrently downregulated DRP1 and FIS1 while enhancing OPA1 expression. Myricetin, a key flavonoid in *M. communis*, is a potent ROS scavenger and stabilizes eNOS activity, thereby increasing nitric oxide bioavailability. NO itself is a known regulator of mitochondrial fusion (36). Thus, *M. communis* likely protects the mitochondrial network through dual mechanisms: direct antioxidant action and NO-mediated fusion promotion.

*Cynara cardunculus* uniquely enhanced fusion gene expression (MFN2, OPA1) without significantly altering fission genes. Cynarin and luteolin, its major bioactive compounds, have been reported to modulate kinase signaling pathways (e.g., MAPK) and inhibit LDL oxidation (35). Our data suggest that *C. cardunculus* primarily promotes mitochondrial reconnection and network repair, rather than inhibiting fragmentation.

Efficient removal of damaged mitochondria via mitophagy is essential to prevent the accumulation of mtROS and subsequent activation of the NLRP3 inflammasome (17, 22). Our RT-qPCR data show that ox-LDL significantly suppressed the expression of key mitophagy regulators: PINK1 (PTEN-induced putative kinase 1), PARKIN (E3 ubiquitin ligase), FUNDC1, and OPTN. This suggests a blockade in both the canonical ubiquitin-dependent pathway (PINK1/PARKIN) and the receptor-mediated pathway (FUNDC1/OPTN).

Herbal treatment differentially restored these pathways. *C. cardunculus* uniquely and robustly upregulated PINK1 (3.1-fold) and PARKIN (2.7-fold) vs. ox-LDL (*p* < 0.01). In the PINK1/PARKIN pathway, PINK1 accumulates on depolarized mitochondria, phosphorylates ubiquitin, and recruits PARKIN, which then ubiquitinates outer membrane proteins, tagging the mitochondrion for autophagic degradation. The transcriptional upregulation of PINK1 and PARKIN by *C. cardunculus* suggests enhanced capacity for ubiquitin-dependent mitophagy. *R. officinalis* and *M. communis* preferentially increased FUNDC1 and OPTN expression. FUNDC1 is a receptor on the outer mitochondrial membrane that directly binds LC3 to initiate mitophagy under hypoxic or stress conditions. OPTN (optineurin) is an autophagy receptor that bridges ubiquitinated cargo to LC3. The upregulation of these genes indicates activation of PINK1/PARKIN-independent, receptor-mediated clearance mechanisms.

While these transcriptional changes are highly suggestive, definitive proof of enhanced mitophagic flux requires functional validation. PINK1 activity is regulated by its accumulation on depolarized mitochondria (a post-translational event), not solely by mRNA levels. Similarly, FUNDC1 activity is controlled by phosphorylation status. Elevated SQSTM1/p62 mRNA, observed in some groups, can indicate either increased autophagic flux or impaired degradation. Therefore, our conclusions represent a molecular hypothesis that must be confirmed via Western blotting (PINK1, PARKIN, LC3-II, p62) and functional flux assays (e.g., using lysosomal inhibitors like bafilomycin A1).

The mitochondrial protection conferred by the extracts is further supported by their effects on endogenous antioxidants: *M. communis* significantly increased CAT expression, and all three extracts restored SOD levels (Figure 4). These enzymes neutralize mtROS, breaking the vicious cycle of oxidative stress that activates the NLRP3 inflammasome. ox-LDL induced a marked upregulation of AKT (RAC-gamma serine/threonine-protein kinase isoform 1) expression, likely as a compensatory survival response to cellular stress. All three extracts normalized AKT levels, supporting cell survival and counteracting mitochondrial apoptosis. All three extracts normalized AKT levels.

ox-LDL upregulated MAPK8 (JNK), a stress-activated kinase that promotes apoptosis and inflammation. All three extracts significantly downregulated MAPK8 (*p* < 0.05). This is consistent with the known ability of polyphenols to inhibit the MAPK pathway (35, 47–49).

Despite this comprehensive mechanistic framework, we must explicitly acknowledge limitations. First, our conclusions regarding mitophagy activation are based exclusively on mRNA expression data. While the transcription of PINK1, PARKIN, FUNDC1, and OPTN is upregulated, mRNA levels do not always correlate with functional protein activity. Post-translational modifications—such as PINK1 accumulation on depolarized mitochondria or phosphorylation of FUNDC1—are key regulators of mitophagy flux and were not assessed here. Mitophagy activation requires confirmation via Western blotting (for PINK1, PARKIN, p62, LC3-II) and functional flux assays (e.g., using lysosomal inhibitors like bafilomycin A1). Second, the simplified HUVEC *in vitro* model does not capture the multicellular complexity of atherosclerotic plaque development, including interactions with macrophages and smooth muscle cells.

## 5 Conclusions

In summary, our study provides a detailed molecular blueprint demonstrating that R. officinalis, C. cardunculus, and M. communis protect HUVECs from ox-LDL-induced injury by: (1) suppressing the excessive fission machinery (DRP1, FIS1, MFF); (2) restoring fusion proteins (MFN2, OPA1); (3) transcriptionally upregulating complementary mitophagy pathways (PINK1/PARKIN and FUNDC1/OPTN); and (4) reinforcing antioxidant defenses (SOD, CAT) while suppressing inflammatory signaling (MAPK8/JNK). The distinct molecular profiles of these extracts suggest potential for synergistic combinations targeting all major nodes of mitochondrial quality control. Future work should focus on: (1) validating transcriptional findings at the protein level and assessing functional mitophagic flux; (2) isolating and characterizing active constituents; (3) validating efficacy in complex *in vitro* and *in vivo* models; and (4) establishing pharmacokinetic parameters to enable clinical translation. Targeting mitochondrial homeostasis with natural products represents a promising, mechanistically grounded approach that warrants further investigation for preventing vascular disease.

## Data Availability

The raw data supporting the conclusions of this article will be made available by the authors, without undue reservation.
